# Analysis of gut microbiota in patients with Williams–Beuren Syndrome reveals dysbiosis linked to clinical manifestations

**DOI:** 10.1038/s41598-023-36704-1

**Published:** 2023-06-16

**Authors:** Federica Del Chierico, Valeria Marzano, Matteo Scanu, Sofia Reddel, Maria Lisa Dentici, Rossella Capolino, Maddalena Di Donato, Iolanda Spasari, Ersilia Vita Fiscarelli, Maria Cristina Digilio, Maria Teresa Abreu, Bruno Dallapiccola, Lorenza Putignani

**Affiliations:** 1 Immunology, Rheumatology and Infectious Diseases Research Area, Unit of Human Microbiome, Bambino Gesù Children’s Hospital, IRCCS, Rome, Italy; 2 Genetics and Rare Diseases Research Division and Medical Genetics Department, Bambino Gesù Children’s Hospital, IRCCS, Rome, Italy; 3 Translational Cytogenomics Research Unit, Bambino Gesù Children’s Hospital, IRCCS, Rome, Italy; 4 Research Unit of Diagnostical and Management Innovations, Bambino Gesù Children’s Hospital, IRCCS, Rome, Italy; 5 Crohn’s and Colitis Center, Division of Digestive Health and Liver Diseases, Department of Medicine, University of Miami, Miller School of Medicine, Miami, FL USA; 6 Scientific Directorate, Bambino Gesù Children’s Hospital, IRCCS, Rome, Italy; 7 Unit of Microbiology and Diagnostic Immunology, Unit of Microbiomics and Immunology, Rheumatology and Infectious Diseases Research Area, Unit of Human Microbiome, Bambino Gesù Children’s Hospital, IRCCS, Rome, Italy

**Keywords:** Genetics, Microbiology, Diseases, Gastroenterology, Medical research, Molecular medicine

## Abstract

Williams–Beuren syndrome (WBS) is a multisystem genetic disease caused by the deletion of a region of 1.5–1.8 Mb on chromosome 7q11.23. The elastin gene seems to account for several comorbidities and distinct clinical features such including cardiovascular disease, connective tissue abnormalities, growth retardation, and gastrointestinal (GI) symptoms. Increasing evidence points to alterations in gut microbiota composition as a primary or secondary cause of some GI or extra-intestinal characteristics. In this study, we performed the first exploratory analysis of gut microbiota in WBS patients compared to healthy subjects (CTRLs) using 16S rRNA amplicon sequencing, by investigating the gut dysbiosis in relation to diseases and comorbidities. We found that patients with WBS have significant dysbiosis compared to age-matched CTRLs, characterized by an increase in proinflammatory bacteria such as *Pseudomonas*, *Gluconacetobacter* and *Eggerthella*, and a reduction of anti-inflammatory bacteria including *Akkermansia* and *Bifidobacterium*. Microbial biomarkers associated with weight gain, GI symptoms and hypertension were identified. Gut microbiota profiling could represent a new tool that characterise intestinal dysbiosis to complement the clinical management of these patients. In particular, the administration of microbial-based treatments, alongside traditional therapies, could help in reducing or preventing the burden of these symptoms and improve the quality of life of these patients.

## Introduction

Williams–Beuren syndrome (WBS) is a multisystem disorder^1^, caused by the deletion of approximately 1.5–1.8 Mb on chromosome 7q11.23 that encompasses 27 genes, including elastin^2^. This syndrome is estimated to occur in approximately one in 7,500 individuals^3^. Elastin deficiency is likely associated with other distinct physical features such as connective tissue abnormalities, the facial “elfin-like” physiognomy, mental retardation, friendly personality, and growth retardation^4,5^. Cardiovascular diseases occur in 80% of patients and are the leading cause of morbidity and mortality^6^. Additional problems are infantile hypercalcaemia, renal tract abnormalities, strabismus, sensory processing impairments (especially hypersensitivity to sounds), premature aging and gastrointestinal (GI) diseases^7^. The main GI symptoms include colic, constipation, gastroesophageal reflux (GERD), abdominal pain of unclear cause, colonic volvulus, diverticular disease, rectal prolapse and celiac disease^8,9^. Infants and children with WBS show a reduced fat mass, with a low weight because of feeding and GI problems^10–12^. However, during early adolescence and adulthood the weight can increase in some subjects to overweight or obesity^13–15^. Alterations in the gut microbiota have been implicated in the pathogenesis of GI and extra-intestinal disorders, including inflammatory bowel disease (IBD), celiac disease, cardiovascular disease, allergy, asthma, metabolic syndrome, obesity and autism spectrum disorders^16–18^. For this reason, we hypothesized that the spectrum of GI manifestations in WBS could be attributed to changes in the gut microbiota. To date, no study has been conducted to examine the gut microbiota in WBS.

Herein, the gut microbiota profiles of patients with WBS were compared with those from healthy subjects. Moreover, we asked whether patients had intestinal dysbiosis and whether dysbiosis had any relationship with specific clinical manifestations and comorbidities.

## Results

### Study population

The WBS study cohort consisted of 26 females and 20 males (Table 1). The median age at time of study participation was 13 years (interquartile range [IQR] = 11.75 years).

**Table 1:** Clinical and anthropometric features of WBS patients.

Features	WBS patients N
Number of subjects	46
Age median (IQR^1^) years	13 (11.75)
Gender (F/M)	26/20
Weight median (IQR) kg	41.25 (40.65)
Height median (IQR) cm	144.75 (29.78)
BMI^2^ median (IQR) kg/m^2^	18.98 (9.31)
Cardiovascular abnormalities (%)	26 (56.5)
Hypertension (%)	19 (41.3)
Hypothyroidism (%)	9 (19.6)
“Elfin-like” facial features (%)	41(89.1)
Motor and/or cognitive impairments (%)	31 (67.4)
Presence of gastrointestinal symptoms (%)	20 (43)
GERD^3^ (%)	12 (24)
Diarrhoea (%)	6 (13)
Constipation (%)	14 (30)
Abdominal pain (%)	7 (15)

^1^Interquartile range; ^2^Body mass index; ^3^Gastroesophageal reflux disease.

Cardiovascular abnormalities were reported for 26 patients. Nineteen patients had hypertension and nine hypothyroidisms. For 31 patients motor and/or cognitive impairments were reported. In the overall cohort, 20 patients experienced GI symptoms of whom 12 specifically had GERD symptoms, 6 patients reported diarrhoea, 14 constipation and 7 abdominal pain. Diabetes was diagnosed in 2 subjects. Forty-one of 46 patients had characteristic facial features of WBS. Lastly, 2 patients followed a hypocaloric diet, and 4 an elimination diet for food intolerance.

### Age and gender do not influence gut microbiota composition

**Figure 1:**
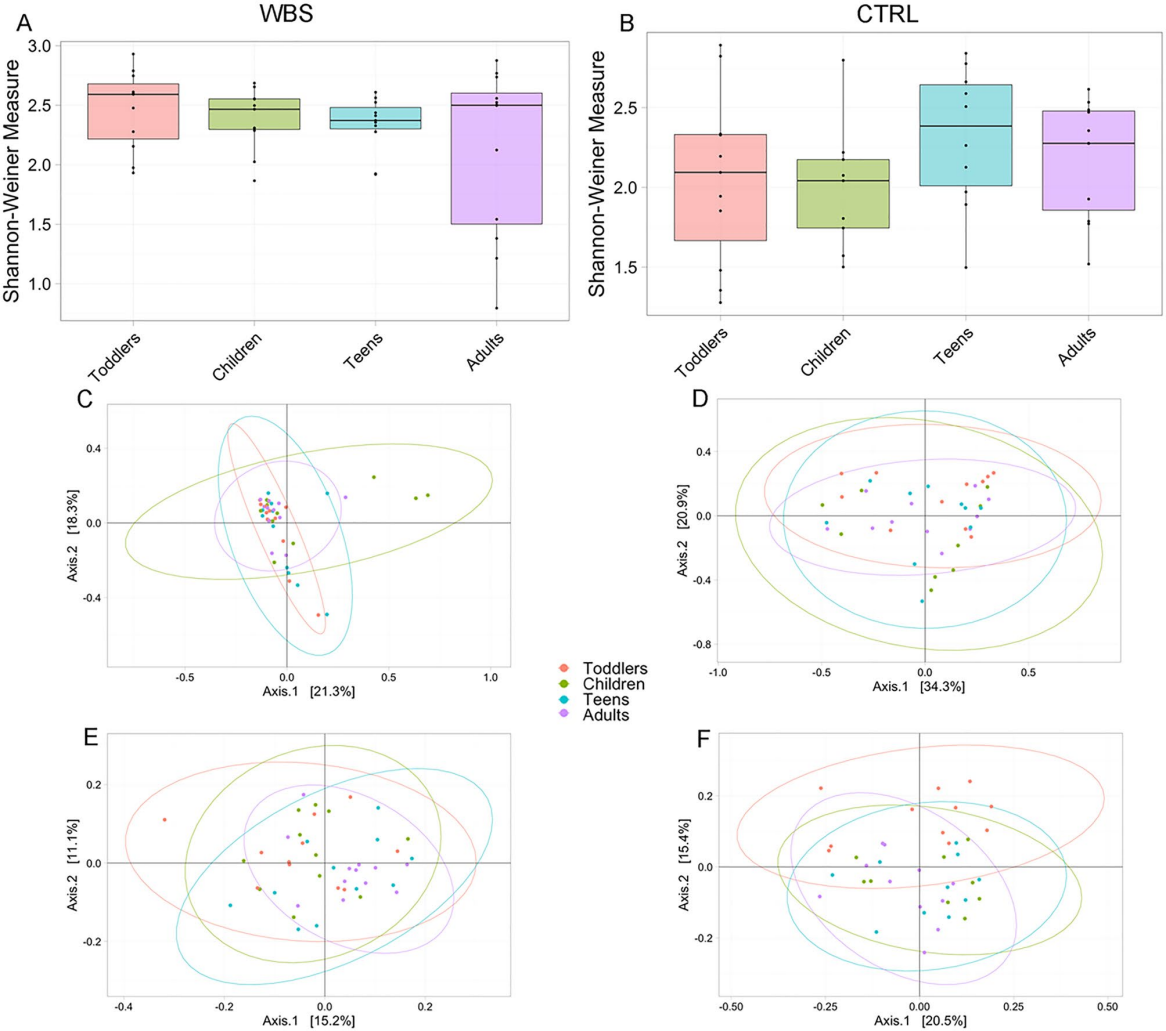
Ecological analyses of WBS and CTRL stratified by age groups. Alpha diversity analysis (**A** and **B**). Box plots show the Shannon-Weiner index of WBS and CTRL groups based on their age’s classes. In box plot the values of median, first and third quartiles, minimum and maximum values of Shannon index for each group are reported. Statistical test is performed by Kruskal–Wallis test (*p*-adj values > 0.05). Beta-diversity analyses. Principal Coordinates Analysis (PCA) plots of Bray Curtis dissimilarity (**C** and **D**) and Unweighted UniFrac phylogenetic distance matrices (**E** and **F**). Each ellipse represents the 95% confidence interval of standard error. The PERMANOVA test applied on Bray–Curtis dissimilarity reveals the absence of a statistically significant dissimilarity amongst age groups (WBS *p* value = 0.11, CTRL *p* value = 0.11). The PERMANOVA test applied on Unweighted UniFrac reveals a statistically significance (WBS *p* value = 0.02, CTRL *p* value = 0.04). The ANOSIM test on Bray–Curtis confirms the similarity amongst the age groups (WBS: *R*-value = 0.02, *p* value = 0.21; CTRL: *R* value = 0.06, *p* value = 0.08). The same analysis performed on Unweighted UniFrac matrices results not statistically significant for WBS (*R* = 0.03; *p* value = 0.09) while statistically significant for CTRLs (*R*-value = 0.09; *p* value = 0.01).

Sequencing analysis of bacterial 16S rRNA V3–V4 regions generated 8,682,610 high-quality reads (average ± standard deviation 92,398.5 ± 57,944.4 reads), after filtering, we had 154 amplicon sequence variants (ASV) at the genus level. To test if age and gender could be confounding factors in gut microbiota profiling analyses, we independently considered the WBS and CTRL groups, stratified for these variables. For the age stratification we identified four age groups^19^: “toddlers”, 1–6 years; “children”, 7–12 years; “teens”, 13–18 years; “adults” over 19 years old. The Wilcoxon test applied on the Shannon-Weiner index (α-diversity) revealed the absence of statistically significant differences amongst age groups for either the WBS or CTRL cohorts (*p*-adj value = 0.67 and *p*-adj value = 0.31, respectively) (Fig. 1A,B).

The β-diversity measured by Bray–Curtis and Unweighted UniFrac algorithms showed low dissimilarity amongst age groups in both WBS and CTRL groups (Fig. 1C,D,E,F).

The β-diversity analyses highlighted the absence of statistically significant differences (PERMANOVA test *p* value > 0.05) amongst subjects stratified for age, for both WBS and CTRL groups. This result was confirmed by the ANOSIM test, that showed the statistically significant similarity amongst subjects (test *p* value < 0.05).

To evaluate the effect of “gender” on gut microbiota ecology, the same statistical approach was applied (data not showed), resulting in the absence of statistically significant differences between male and female, on α- and β-diversity. The multivariate analysis applied on the microbial genus matrices of the gut microbiota composition (Figure S1, panel A–D), confirmed the results obtained on α- and β-diversity, excluding the both the variables “age” and “gender” as confounding factors.

### WBS influences the gut microbiota composition

**Figure 2:**
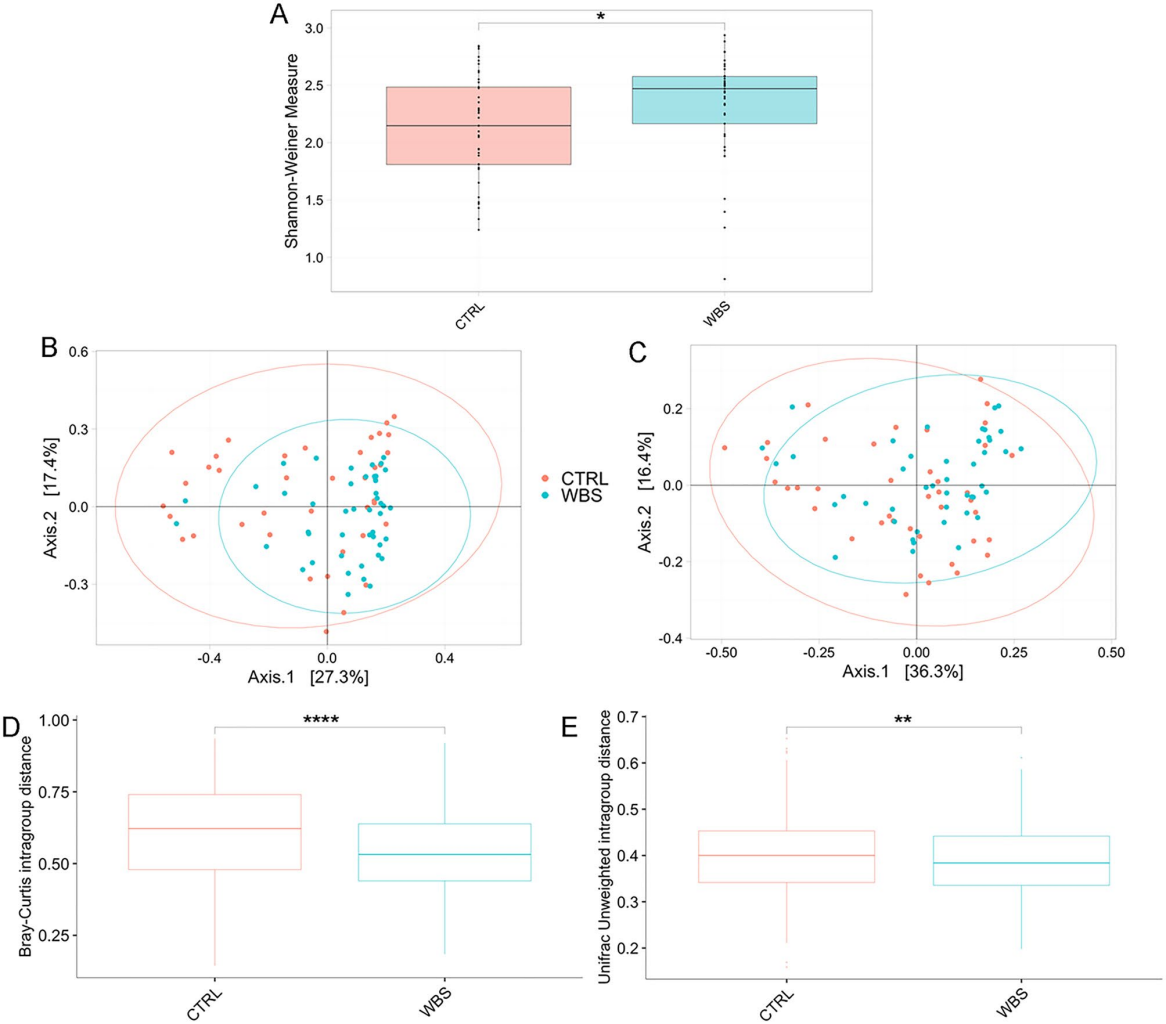
Ecological analyses of WBS and CTRL. Alpha diversity analysis (**A**). Box plots show the Shannon-Weiner index of WBS and CTRL. In box plot the values of median, first and third quartiles, minimum and maximum values of Shannon index for both groups are reported. Beta-diversity analyses. Principal Coordinates Analysis (PCA) plots of Bray Curtis dissimilarity (**B**) and Unweighted UniFrac phylogenetic distance matrices (**C**). Each ellipse represents the 95% confidence interval of standard error. The PERMANOVA and ANOSIM tests applied on β-diversity matrices reveal the statistically significant dissimilarity between WBS and CTRLs (Bray–Curtis matrix: PERMANOVA *p* value = 0.002; ANOSIM: R value = 0.10, *p* value = 0.001; unweighted UniFrac matrix: PERMANOVA *p* value = 0.001; ANOSIM R value = 0.14; *p* value = 0.001). Intragroup distances calculation. Box plots of intragroup distances calculated on (**D**) Bray–Curtis and (**E**) Unweighted UniFrac distances. Statistically significant comparisons by Wilcoxson test are indicated by asterisk (*p-adj ≤ 0.05; **p-adj ≤ 0.01; ****p-adj ≤ 0.0001).

Analysis of gut microbiota of WBS patients compared to CTRLs demonstrated an increase of the α-diversity Shannon index (*p*-adj ≤ 0.05) (Fig. 2A).

Beta diversity analyses, performed by Bray–Curtis and Unweighted UniFrac algorithms, revealed statistically significant differences between the two groups (Fig. 2B,C). The intragroup distance calculation highlighted a reduced distance amongst WBS with respect to the CTRLs, pointing to more similarity amongst WBS than to CTRLs (*p*-adj ≤ 0.05) (Fig. 2D,E).

**Figure 3:**
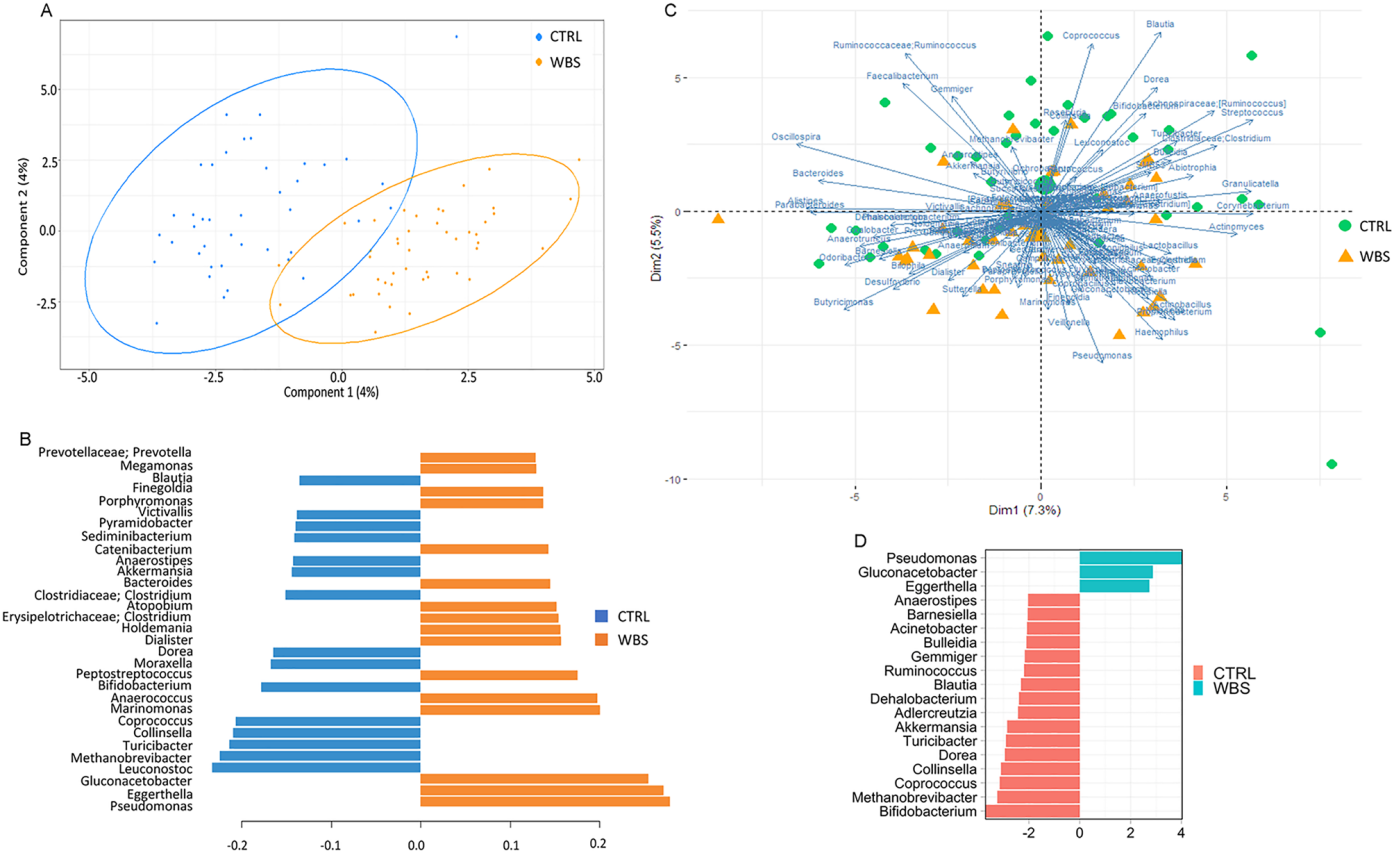
Compositional analyses at the genus level of WBS and CTRL. (**A**) Partial least squares discriminant analysis (PLS-DA) plot; (**B**), plot of loading variables, filtered for loading coefficient > 0.1. The Root Mean Square Error (RMSE) = 0.24 indicates a good accuracy in classification’s prediction; (**C**), Principal component analysis (PCA) plot. More the loadings are distant from the origin, more they influence the model. The loadings separated by a small angle show a positive correlation; the loadings separated by a large angle have a negative correlation, and those with a right angle indicate no correlation. (**D**), Univariate ANCOM-BC plot.

We applied multivariate approaches, based on PLS-DA and PCA, to explore the ability of the microbial composition at the genus level to represent the sample set. The PLS-DA results are plotted in Fig. 3A. The model shows good accuracy in classification prediction with a very low Root Mean Square Error (RMSE) value. In panel B, we report the loading variables predicting the model (Fig. 3B). The PCA biplot reports PC scores of samples (dots and triangles) and variable loadings (vectors) explaining the model (Fig. 3C). Bacterial abundance in WBS and CTRLs was also investigated by the univariate approach. At the phylum and family levels, some differences in gut microbiota composition were apparent (Figure S2A,B; Table S1). At the genus level, we found an increase of *Pseudomonas*, *Gluconacetobacter,* and *Eggerthella*, and a decrease of *Anaerostipes, Barnesiella, Acinetobacter, Bulleidia, Gemmiger, Ruminococcus, Blautia, Dehalobacterium, Adlercreutzia, Akkermansia, Turicibacter, Dorea, Collinsella, Coprococcus Methanobrevibacter,* and *Bifidobacterium* was found in WBS (Fig. 3D). Both multivariate and univariate approaches confirmed the importance of *Pseudomonas*, *Gluconacetobacter*, *Eggerthella* to describe the gut microbiota in WBS, and *Anaerostipes*, *Blautia*, *Akkermansia*, *Turicibacter*, *Dorea*, *Collinsella*, *Methanobrevibacter* and *Bifidobacterium* in CTRLs.

### Functional analysis of the microbiome discriminates WBS from CTRLs

**Figure 4:**
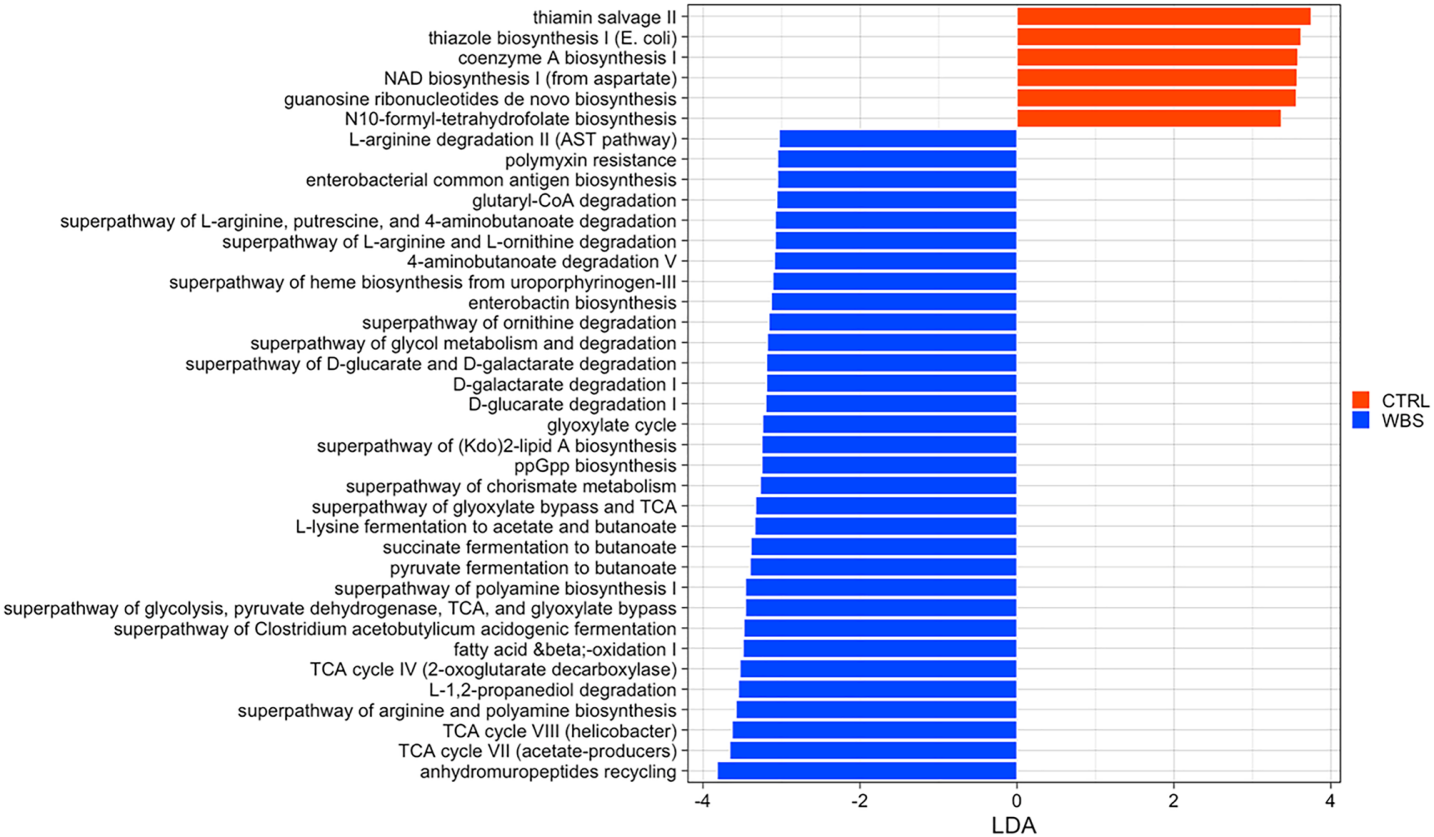
Microbial functional profiling. Linear discriminant analysis (LDA) effect size (LEfSe) was performed on the PICRUSt2 predicted biochemical pathways matrix. The reported pathways were filtered for statistically significance and LDA ± 3.0.

By PICRUSt analysis, 45 biochemical pathways were differentially associated to WBS and 6 to CTRLs (Table S2, Fig. 4). The majority of the pathways associated to WBS belonged to the metabolism pathways; in particular, carbohydrate metabolism (17), amino acid metabolism (11), xenobiotic biodegradation and metabolism (6), metabolism of cofactors and vitamins (3), glycan biosynthesis and metabolism (2), lipid metabolism (2), metabolism of terpenoids and polyketides (1), biosynthesis of other secondary metabolites (1). Two others pathways, belonged to cellular processes and environmental information processing. The pathways associated with CTRLs belong to cofactors and vitamin metabolism (3), amino acid metabolism (1), nucleotide metabolism (1), energy metabolism (1).

### Co-occurrence patterns are reduced in WBS gut microbiota

The correlation network analysis identifies complex interactions between bacteria. Network analysis highlighted the decrease in number of co-occurrence nodes in WBS (134 nodes interconnected by 285 edges) compared to CTRLs (137 nodes linked by 529 edges). In particular, the WBS gut microbiota demonstrated 6 major networks (Figure S3), while the CTRLs microbiota showed an increase of both co-occurrence and co-exclusion patterns, establishing 10 major nodes (Figure S4).

### Clinical and anthropomorphic features of WBS influences gut microbiota composition

The PLS-DA approach was applied to WBS patients to investigate the ability of gut microbiota to classify individuals on the basis of clinical and weight‐related features. By these analyses, we obtained low RMSE values, indicating the accuracy of these models (Figure S5).

**Figure 5:**
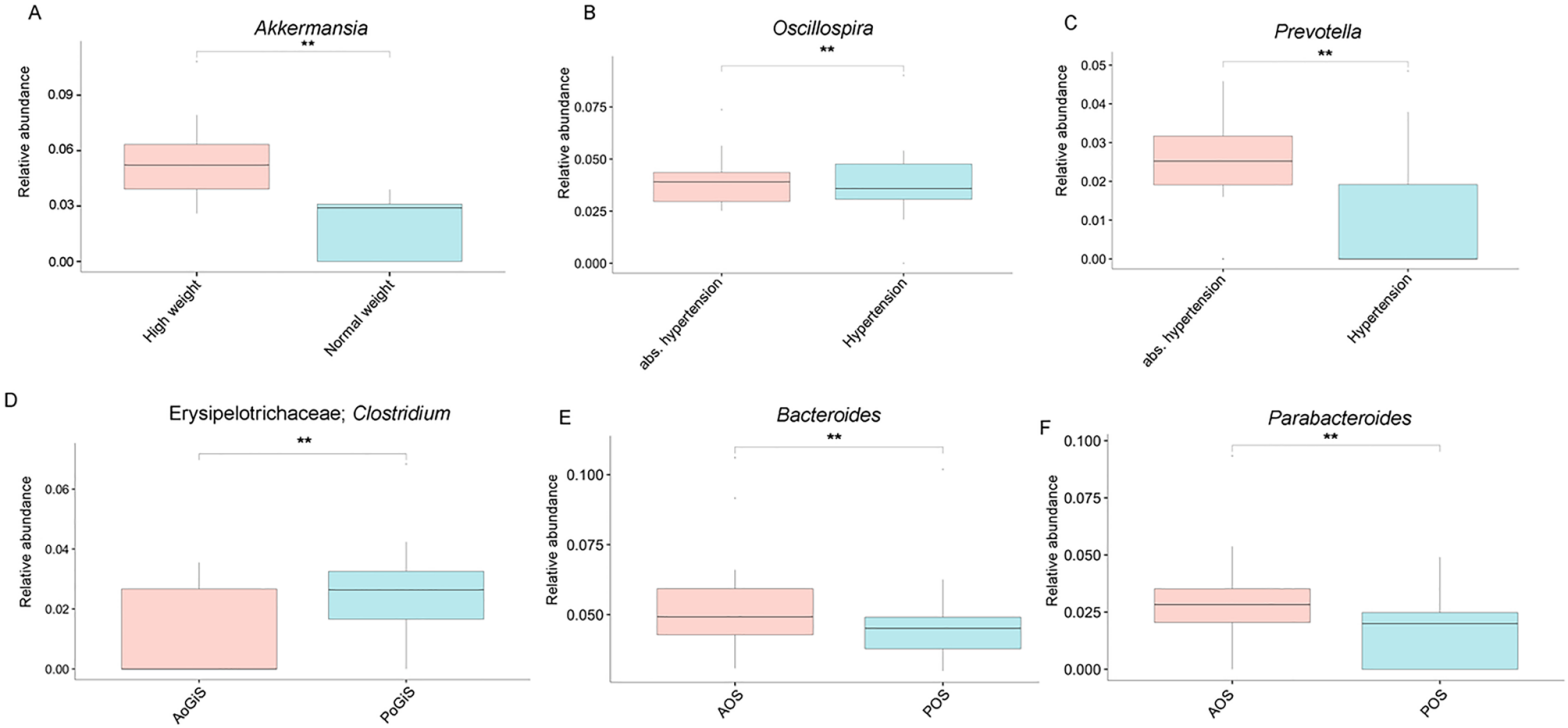
Compositional analyses at genus level of patients stratified for weight‐related and clinical features. Box plots indicate the median abundances and interquartile ranges of the taxa resulted statistically significant by ANCOM-BC test (*p-adj ≤ 0.05; **p-adj ≤ 0.01;). (**A**) comparisons between WBS normal weight and high weight; (**B** and **C**) comparisons between patients stratified for the presence of hypertension or not (abs. hypertension); (**D**) for absence (AoGiS) or presence of gastrointestinal symptoms (PoGiS) (constipation or diarrhoea); (**E** and **F**) for absence (AOS) or presence of at least 1 gastrointestinal symptom (POS) (gastroesophageal reflux disease, diarrhoea, constipation, abdominal pain).

To investigate the role of gut microbiota in the body weight alteration during growth, we compared WBS patients ≤ 12 years old with low weight with those with normal weight, and the WBS > 12 years old with increased weight with those with normal weight. Comparison between normal and low weight did not reveal any significant differences in gut microbiota composition, while comparison between normal with high weight showed that *Akkermansia* was increased in the latter (Fig. 5A).

By stratifying patients for presence/absence of hypertension, we found a decrease in *Oscillospira* and *Prevotella* in hypertensive patients (Fig. 5B,C). Comparison between patients with or without cardiovascular abnormalities did not reveal any significant difference in gut microbiota composition. Last, we considered the relationship between GI symptoms and the microbiota. We dichotomized subjects suffering for diarrhoea or constipation (presence of GI symptoms [PoGiS] group) and those without symptoms (absence of GI symptoms [AoGiS]), and found a decrease in *Clostridium* (Erysipelotrichaceae) in the latter group (Fig. 5D). Finally, comparing patients with the presence of at least one GI symptom (25/46 patients) (POS) including GERD, diarrhoea, constipation, or abdominal pain versus those without symptoms (AOS), a decrease of *Bacteroides* and *Parabacteroides* in the POS group was apparent (Fig. 5E,F). These data support the presence of specific taxa associated with GI manifestations.

## Discussion

We performed the first explorative analysis of gut microbiota in WBS patients compared with that of age- and gender-matched healthy subjects. The strengths of the current study include the large number of WBS patients we studied relative to the rarity of this condition and our ability to match these with healthy children and adolescents from a similar region to have a clear idea of the microbiome in these patients relative to population controls. In addition, we investigated the WBS gut microbiota dysbiosis in relation to clinical features and comorbidities because we had developed a survey that captured a spectrum of clinical manifestations in these patients that have an important impact on quality of life. We found an increase in bacterial diversity of gut microbiota in WBS patients compared with CTRLs. Generally, low complexity of the microbial community, or a reduced biodiversity, is associated with a dysbiotic condition^20^. In the case of WBS, the dysbiosis is not reflected in a decrease in species richness and instead is reflected in a different microbiota phenotype and its function. Johnson and Burnett debated on the significance of the ecological dynamics of gut microbial communities, showing that diversity and stability may not always be concomitant^21^. Moreover, the proposed model of Coyte et al., predicted that high species diversity leads to unstable microbiome communities^22^. However, there is not a full agreement on the definition of dysbiosis and its clinical implications^23^. Thus, dysbiosis cannot be considered either a disease or a symptom, but rather an association with a disease or a symptom and should be explored in the context of a well-matched control group as we have in the current study^23^.

From an ecological point of view, β-diversity analyses show a distinct microbiota profile in WBS, with a reduction of the intragroup distance means there is more similarity among these samples with respect to CTRLs. This was apparent by comparing the α-diversity results of the two cohorts stratified by age. Although age did not influence the gut microbiota composition, the Shannon index value was more uniform amongst WBS compared to CTRLs, pointing to a more important impact of the “disease fingerprint” on the patients’ microbiota rather than age.

Interestingly, in spite of an increase in diversity, the WBS gut microbiota was enriched in inflammatory bacteria (Proteobacteria [newly renamed Pseudomonadota]) and reduced in beneficial phyla, including Verrucomicrobia (newly renamed Verrucomicrobiota), Actinobacteria (newly renamed Actinomycetota), and Firmicutes (newly renamed Bacillota). Some evidence has shown that, during gut inflammation, obligate anaerobes belonging to the Bacteroidetes (newly renamed Bacteroidota) and Firmicutes phyla decrease, whereas Proteobacteria increases to become the most prominent phylum in the gut^24^. The blossom of Proteobacteria is largely due to Gammaproteobacteria outgrowth, including Pseudomonadaceae (*Pseudomonas*) and Enterobacteriaceae families^24,25^. In our study, we observed in WBS patients an increase of *Pseudomonas* and *Gluconacetobacter,* belonging to Proteobacteria phylum, supporting the role of these pathobionts in GI inflammation in these subjects. This evidence is also confirmed by an increase in the enterobacterial antigen biosynthesis pathway based on PICRUSt2, in WBS.

*Eggerthella* was also increased in the patient group. Interestingly, a similar increase was reported in the gut of adults with COVID-19^26–28^ and seems to induce colitis through abnormal activation of Th17 pathways in patients with IBD^28^. Thus, it is conceivable that through an immune-mediated mechanism, *Eggerthella* could interfere with gut integrity, making the patients more susceptible to the invasion of pathogens, like SARS-CoV-2 virus^29^.

On the other hand, we observed a decrease of short chain fatty acids (SCFAs) producers, like *Anaerostipes, Ruminococcus, Blautia, Coprococcus*, *Bifidobacterium, Barnesiella* and *Akkermansia*^30–32^. These bacteria have anti-inflammatory properties and their decrease could contribute to functional dysbiosis. In particular, the reduction of *Bifidobacterium* has been associated to intestinal dysbiosis^33^. Moreover, *Akkermansia* actively stimulates host mucin production, thus increasing mucus layer thickness and, hence, improving gut-barrier function^34^. In general, *Akkermansia* is associated with a healthy GI tract, and its abundance was reported as inversely related with several diseases, such as type 1 and type 2 diabetes, IBD and obesity^35,36^. In the present study, *Akkermansia* was associated with increased weight in WBS patients. Despite the aforementioned anti-inflammatory properties, some studies have reported also an increase of *Akkermansia* in diverticular disease^37–39^. Diverticulosis of the sigmoid is rather common in adults with WBS^40^, while it is rare in paediatric patients^41^. We found the *Akkermansia* overgrowth in high weight older patients, possibly as the result of a homeostatic response against inflammation or because an underlying undiagnosed diverticulosis. Under physiological conditions, the eubiotic gut microbiota shows complex interacting and communicating networks^42^. These cooperative or competitive microbial interactions play a basic role in gut microbiota stability, resistance and resilience, maintaining a dynamic equilibrium required to counteract the perturbations occurred in some pathological conditions^43,44^. Additional evidence of dysbiosis in the WBS gut microbiota is represented in the correlation network analysis, demonstrating poor microbial connections with respect to CTRLs, and thus a less diverse ecological niche. These data suggest an additional level at which the microbiota in WBS patients is dysfunctional.

The functional profile prediction shows a higher number of pathways in WBS, most of which belonging to amino acid and carbohydrate metabolism. In particular, we found an increase of the pathways involved in the polyamine (PA) biosynthesis, starting from the degradation of L-arginine and L-ornithine, and from the production of 4-aminobutanoate (also named gamma-aminobutyric acid [GABA]). PAs are involved in several important cellular processes and their dysregulation can affect growth, aging and a number of diseases, such as cancer, neurodegeneration and metabolic disorders^45,46^. The degradation of GABA and the fermentation of lysin, succinate and pyruvate produce butyrate, which has critical physiological effects on several organs, including the brain^47^; together with other SCFAs these affect the glial cell morphology and functions. SCFAs modulate also the levels of neurotrophic factors, contribute to the biosynthesis of serotonin, and improve neuronal homeostasis and function^48^. WBS patients show mild to moderate mental retardation, overfriendliness, and empathy^49^, and one can speculate that interaction of SCFAs with the gut-brain pathways could directly or indirectly affect mood, emotional and cognitive condition.

We found an increase in tricarboxylic acid cycle (TCA) cycle pathways in WBS. Wian and co-workers demonstrated a positive correlation between the intestinal concentration of TCA intermediates (e.g., citric, fumaric, malic and succinic acids) and the host energy and lipid metabolism^50^. In addition, it has been shown that in obese patients there is a positive correlation between an increased susceptibility to cardiovascular disease and the blood levels of TCA intermediates released by gut microbiota^51^, supporting a link between the increase of these microbial pathways and cardiovascular comorbidities in WBS.

Diarrhoea or constipation are common in WBS individuals^52,53^. In our study, *Clostridium,* a member of the Erysipelotrichaceae family, was increased in patients suffering of these symptoms. These members are medically important anaerobes, both commensals and pathogens of the gut^54^, and some of them are involved in antibiotic-associated diarrhoea^55^. By considering the entire spectrum of GI symptoms, we identified the reduction of *Bacteroides* and *Parabacteroides* in the patients with at least one of these symptoms*. Bacteroides* and *Parabacteroides*, two closely related commensals are highly abundant in the human gut^56^ and are decreased in patients with intestinal inflammation^57,58^, in agreement with the present observations. Finally, we found a link between gut microbiota dysbiosis and hypertension, more specifically a decrease of *Oscillospira* and *Prevotella* in hypertensive patient. This has been described in mouse models^59^.

Although our results are novel and promising, the study has some limitations. Even if this is a large sample size by WBS standards, a larger sample size is required to validate the compositional gut microbiota profile associated with WBS especially as related to clinical features and comorbidities. In addition, larger cohorts including narrow age ranges should be enrolled to avoid possible confounding effects on gut microbiota evaluation. Finally, metabolome experiments could help in determining the functional profile of host and gut microbiota and its role in WBS clinical symptoms. Future longitudinal clinical trials could evaluate the role of specific probiotic administration in the development/amelioration of WBS related symptoms.

In conclusion, our study demonstrated a distinctive dysbiotic gut microbiota in WBS patients that is associated with GI symptoms, hypertension, and high BMI. These results could offer new perspectives for patient management, including some of the comorbidities in this genetic condition. In the future, we plan to confirm and validate these preliminary results on a larger patient cohort. Nevertheless, present results suggest the possibility to test specific targeted microbial treatments, such as pre/probiotics and faecal microbiota transplantation, alongside traditional therapies, for reducing or preventing some symptoms and improving the quality of life of these patients.

## Methods

### Patients

A cohort of 46 consecutive patients were enrolled at the Medical Genetics Unit of Children’s Hospital Bambino Gesù in Rome. The diagnosis of WBS was established by detecting recurrent heterozygous deletion at chromosome 7q11.23. The clinical diagnosis of patients was confirmed either by deletion analysis through fluorescence in situ hybridization (FISH) targeted to the 7q11.23 region^60^, or by chromosomal microarray (CMA), that can detect the 7q11.23 deletion^61,62^. Medical histories were obtained by direct interview of subjects and parents to detect major medical complications connected to the syndrome.

Age, gender, weight, height, GERD, GI symptoms (abdominal pain, diarrhoea, or constipation), neuropsychiatric and cardiac signs and symptoms were recorded for each patient at the time of enrolment (Table 1). BMI was calculated for all subjects, based on Centers for Disease Control and Prevention (CDC) calculator (https://www.cdc.gov/healthyweight/bmi/calculator.html).

For each clinical variable, we calculated the frequency of the occurrence in our WBS cohort. By this approach, patients were stratified in groups (presence or absence of the clinical manifestation) to perform the uni- and multivariate statistical analyses, described below.

A cohort of 41 gender- and age-matched healthy subjects (controls, CTRLs) were enrolled during an epidemiological survey carried out at the Human Microbiome Unit of Bambino Gesù Children’s Hospital (BBMRI Human Microbiome Biobank, OPBG) to generate a reference biobank of samples from healthy subjects. The exclusion criteria were: a family history of chronic or autoimmune diseases, BMI ≤ 18.4 or BMI > 24.9, GI diseases. Moreover, we excluded subjects who have taken antibiotics and/or pre/probiotics in the previous two months before recruitment.

The study was approved by the Ethical Committee of the Bambino Gesù Children's Hospital, IRCCS (protocol No. 2590_OPBG_2021; healthy subjects: protocols No. 1113_OPBG_2016 and No.2839_OPBG_2022) and was conducted in accordance with the Principles of Good Clinical Practice and the Declaration of Helsinki. Written informed consent was obtained from either parents or legal representative of the children. From each subject in these cohorts, a single faecal sample was collected and stored at − 80 °C until further analyses.

### 16S rRNA-based gut microbiota profiling

From 200 mg of stools, DNA was extracted by QIAmp Fast DNA Stool mini kit (Qiagen, Germany), following the manufacturer’s instructions. The V3–V4 variable region (~ 460 bp) of 16S rRNA was amplified following the MiSeq rRNA Amplicon Sequencing protocol (Illumina, San Diego, CA). The DNA amplifications were set up using a 2 × KAPA Hifi HotStart ready Mix (KAPA Biosystems Inc., Wilmington, MA, USA) following the manufacturer’s protocol. The DNA amplicons were cleaned up by AMPure XP beads (Beckman Coulter Inc., Beverly, MA, USA). Illumina Nextera adaptor-primers were used to barcode each sample. The final library was quantified by Quant-iT™ PicoGreen® dsDNA Assay Kit (Thermo Fisher Scientific, Waltham, MA). Samples were sequenced on an Illumina MiSeq™ platform according to the manufacturer’s specifications to generate paired-end reads of 300 base-length [ 35].

### Bioinformatic analysis

A total of 172 fastq files were imported into QIIME2 v2022.2^63^. The Quality Check (QC) was performed: all samples had a sample depth greater than 1000 reads and the minimum phred score value was greater than 20. By means of DADA2^64^, paired-end reads were filtered from chimeras and assembled into ASVs with a cut off of 99% similarity. Using a Naive Bayes classifier, the taxonomy of each sequence was analysed against the Greengenes 16S rRNA database (Version 13.8, https://greengenes.secondgenome.com)^65^. Sequences labelled with Chloroplast, Mitochondria, Eukaryota were filtered. ASV table was filtered out retaining the ASV present in at least the 25% of the total samples. Moreover, a filter based on the ASV relative abundance, retaining ASV with relative abundance > 0.01, was applied. The phylogenetic tree was built with the phylogeny align-to-tree-mafft-fasttree method, which is based on a de novo approach called de novo phylogenetic tree^66^.

### Statistical analyses

Reads were rarefied to the low number of sequences in a sample for the α- and β-diversity analyses, performed by Phyloseq package v1.38.0^67^. Prior to conduct comparison statistical analyses, ASVs abundances from each sample were normalized with the Cumulative Sum Scaling (CSS) method^68^. Kruskal–Wallis, Mann–Whitney, tests were applied for α-diversity comparisons. We used both PERMANOVA and ANOSIM approaches to test the statistically significance of β-diversity results. The PERMANOVA test checks the null hypothesis of no differences amongst groups in the positions of the centroid and space dispersion of each group (dissimilarity measure)^69^. The ANOSIM test checks the null hypothesis that the similarity between groups is greater than or equal to the similarity within the groups (similarity measure)^70^. The analysis of composition of microbiomes with bias correction (ANCOM-BC) test was applied to ASV relative abundance, grouped at different taxonomy levels^71^.

Based on the MetaCyc open-source database^72^ , PICRUSt2 software was used to predict functional pathways^73^. To identify statistically significant biochemical pathways, linear discriminant analysis (LDA) effect size (LEfSe) analysis was used (α value of 0.05 and a logarithmic LDA score threshold of 3.0)^74^. Correlations between microorganisms were performed by using Spearman’s correlation test by means of Hmisc package v4.7–1, and networks were obtained with igraph package v1.3.5. All p-values were adjusted for multiple testing with the Benjamini–Hochberg procedure.

The principal component analysis (PCA) on the scaled data, obtained on the bacterial genus matrix, was performed with the *prcomp* function and the score plot and the biplot were visualized through the *Factoextra package of R.*

For the Partial Least Squares-Discriminant Analysis (PLS-DA), the *plsda* function of the *MixOmics* package was used and the algorithm was a regression model. To evaluate the performance of the model and the overfitting phenomenon, a cross validation was performed with 200 random permutations, using the *Bioconductor ropls package*. The score plots and loading plots were generated through the *mixOmics package*, representing in the *Loadingplot* only the taxa with a loading *coefficient between 0.1 and 0.3 in absolute value.*

### Ethics approval and consent to participate

The study was approved by the Ethical Committee of the Bambino Gesù Children's Hospital, IRCCS (protocol No. 2590_OPBG_2021; healthy subjects: protocols No. 1113_OPBG_2016 and No.2839_OPBG_2022) and was conducted in accordance with the Principles of Good Clinical Practice and the Declaration of Helsinki. Written informed consent was obtained from either parents or legal representative of the children.

## Supplementary Information

Supplementary Information.

## Data Availability

All raw sequences have been archived in NCBI database: PRJNA934831 and PRJNA280490 (https://www.ncbi.nlm.nih.gov/bioproject).
