# Supplementary material for: Analysis of gut microbiota in patients with Williams–Beuren Syndrome reveals dysbiosis linked to clinical manifestations
Source: Sci Rep. 2023 Jun 16;13:9797. doi: 10.1038/s41598-023-36704-1 (PMC10275996; doi:10.1038/s41598-023-36704-1)
Supplement: Supplementary file 1 — Supplementary Information. [file 41598_2023_36704_MOESM1_ESM.docx]

**Supplementary materials**

**Supplementary Table S1.** Differential abundance analyses were performed by ANCOM-BC (analysis of compositions of microbiomes with bias correction) test at the phylum, family and genus levels of the taxonomy. Enriched group, effect size (ef_W, log fold change), p-value, and Benjamini-Hochberg adjusted p-value (p-adj) are provided.

| **Phylum** | **enriched group** | **ef_W** | **p value** | **p-adj** |
| --- | --- | --- | --- | --- |
| p__Proteobacteria | WBS | 3.19 | 1.41E-03 | 1.41E-03 |
| p__Actinobacteria | CTRL | -4.29 | 1.82E-05 | 5.45E-05 |
| p__Euryarchaeota | CTRL | -4.14 | 3.50E-05 | 7.00E-05 |
| p__Firmicutes | CTRL | -6.05 | 1.48E-09 | 8.89E-09 |
| p__Tenericutes | CTRL | -3.50 | 4.70E-04 | 5.64E-04 |
| p__Verrucomicrobia | CTRL | -3.56 | 3.69E-04 | 5.53E-04 |
| **Family** | **enriched group** | **ef_W** | **p value** | **p-adj** |
| f__Enterobacteriaceae | WBS | 3.96 | 7.65E-05 | 2.68E-04 |
| f__Pseudomonadaceae | WBS | 4.25 | 2.16E-05 | 1.51E-04 |
| f__Bifidobacteriaceae | CTRL | -3.71 | 2.07E-04 | 5.79E-04 |
| f__Christensenellaceae | CTRL | -4.75 | 2.00E-06 | 2.80E-05 |
| f__Coriobacteriaceae | CTRL | -2.02 | 4.29E-02 | 4.29E-02 |
| f__Dehalobacteriaceae | CTRL | -2.39 | 1.71E-02 | 1.99E-02 |
| f__Lachnospiraceae | CTRL | -2.90 | 3.70E-03 | 4.71E-03 |
| f__Leuconostocaceae | CTRL | -3.00 | 2.74E-03 | 4.71E-03 |
| f__Methanobacteriaceae | CTRL | -3.44 | 5.75E-04 | 1.34E-03 |
| f__Moraxellaceae | CTRL | -2.34 | 1.93E-02 | 2.08E-02 |
| f__Oxalobacteraceae | CTRL | -2.94 | 3.31E-03 | 4.71E-03 |
| f__Turicibacteraceae | CTRL | -2.93 | 3.39E-03 | 4.71E-03 |
| f__Verrucomicrobiaceae | CTRL | -2.90 | 3.70E-03 | 4.71E-03 |
| **Genus** | **enriched group** | **ef_W** | **p value** | **p-adj** |
| g__Eggerthella | WBS | 2.74 | 0.0061 | 0.0111 |
| g__Gluconacetobacter | WBS | 2.88 | 0.0040 | 0.0086 |
| g__Pseudomonas | WBS | 4.04 | 0.0001 | 0.0005 |
| g__Acinetobacter | CTRL | -2.08 | 0.0378 | 0.0420 |
| g__Adlercreutzia | CTRL | -2.43 | 0.0153 | 0.0254 |
| g__Akkermansia | CTRL | -2.86 | 0.0043 | 0.0086 |
| g__Anaerostipes | CTRL | -2.03 | 0.0421 | 0.0421 |
| g__Barnesiella | CTRL | -2.05 | 0.0405 | 0.0421 |
| g__Bifidobacterium | CTRL | -3.72 | 0.0002 | 0.0013 |
| g__Blautia | CTRL | -2.31 | 0.0208 | 0.0297 |
| g__Bulleidia | CTRL | -2.10 | 0.0358 | 0.0420 |
| g__Collinsella | CTRL | -3.10 | 0.0019 | 0.0064 |
| g__Coprococcus | CTRL | -3.15 | 0.0017 | 0.0064 |
| g__Dehalobacterium | CTRL | -2.39 | 0.0168 | 0.0259 |
| g__Dorea | CTRL | -2.94 | 0.0032 | 0.0086 |
| g__Gemmiger | CTRL | -2.16 | 0.0308 | 0.0385 |
| g__Methanobrevibacter | CTRL | -3.24 | 0.0012 | 0.0059 |
| g__Ruminococcus | CTRL | -2.19 | 0.0286 | 0.0381 |
| g__Turicibacter | CTRL | -2.90 | 0.0037 | 0.0086 |

**Supplementary Table S2.** Biochemical pathways associated to WBS (45) and CTRL (6) obtained by PICRUST analysis.

| **Metabolic classes** | **pathways** | **statistic** | **p.value** | **p.adj** | **CTRL** | **WBS** | **LDA** | **direction** |
| --- | --- | --- | --- | --- | --- | --- | --- | --- |
|  |  |  |  |  |  |  | **score** |  |
| Metabolism; Metabolism of cofactors and vitamins | coenzyme A biosynthesis I | 13.03 | 0.0003 | 0.0026 | 75263 | 67742 | 3.58 | CTRL |
| Metabolism; Metabolism of cofactors and vitamins | thiazole biosynthesis I (E. coli) | 15.72 | 0.0001 | 0.001 | 57141 | 48892 | 3.62 | CTRL |
| Metabolism; Metabolism of cofactors and vitamins | thiamin salvage II | 21.92 | 0.0000 | 0.0002 | 62732 | 51434 | 3.75 | CTRL |
| Metabolism; Amino acid metabolism | NAD biosynthesis I (from aspartate) | 15.44 | 0.0001 | 0.0011 | 61641 | 54232 | 3.57 | CTRL |
| Metabolism; Nucleotide metabolism | guanosine ribonucleotides de novo biosynthesis | 14.84 | 0.0001 | 0.0012 | 80546 | 73212 | 3.56 | CTRL |
| Metabolism; Energy metabolism | N10-formyl-tetrahydrofolate biosynthesis | 12.6 | 0.0004 | 0.003 | 68951 | 64254 | 3.37 | CTRL |
| Metabolism; Amino acid metabolism | superpathway of arginine and polyamine biosynthesis | 12.91 | 0.0003 | 0.0027 | 13540 | 21213 | 3.58 | WBS |
| Metabolism; Amino acid metabolism | superpathway of polyamine biosynthesis I | 12.66 | 0.0004 | 0.003 | 9035 | 14862 | 3.46 | WBS |
| Metabolism; Amino acid metabolism | superpathway of chorismate metabolism | 13.84 | 0.0002 | 0.0019 | 3641 | 7404 | 3.27 | WBS |
| Metabolism; Amino acid metabolism | superpathway of ornithine degradation | 21.31 | 0.0000 | 0.0002 | 1162 | 4066 | 3.16 | WBS |
| Metabolism; Amino acid metabolism | superpathway of L-arginine and L-ornithine degradation | 19.79 | 0.0000 | 0.0002 | 1226 | 3652 | 3.08 | WBS |
| Metabolism; Amino acid metabolism | superpathway of L-arginine, putrescine, and 4-aminobutanoate degradation | 19.79 | 0.0000 | 0.0002 | 1226 | 3652 | 3.08 | WBS |
| Metabolism; Amino acid metabolism | L-arginine degradation II (AST pathway) | 21.65 | 0.0000 | 0.0002 | 863 | 3006 | 3.03 | WBS |
| Metabolism; Amino acid metabolism | phenylacetate degradation I (aerobic) | 21.15 | 0.0000 | 0.0002 | 110 | 858 | 2.57 | WBS |
| Metabolism; Amino acid metabolism | L-histidine degradation II | 15.89 | 0.0001 | 0.001 | 539 | 1267 | 2.56 | WBS |
| Metabolism; Amino acid metabolism | ectoine biosynthesis | 27.72 | 0.0000 | 6E-05 | 13 | 699 | 2.54 | WBS |
| Metabolism; Amino acid metabolism | 4-hydroxyphenylacetate degradation | 23.86 | 0.0000 | 0.0001 | 99 | 732 | 2.5 | WBS |
| Metabolism; Carbohydrate metabolism | anhydromuropeptides recycling | 13.6 | 0.0002 | 0.0021 | 20753 | 33940 | 3.82 | WBS |
| Metabolism; Carbohydrate metabolism | TCA cycle VII (acetate-producers) | 19.48 | 0.0000 | 0.0002 | 9918 | 19149 | 3.66 | WBS |
| Metabolism; Carbohydrate metabolism | TCA cycle VIII (helicobacter) | 14.77 | 0.0001 | 0.0012 | 11032 | 19564 | 3.63 | WBS |
| Metabolism; Carbohydrate metabolism | L-1,2-propanediol degradation | 15.41 | 0.0001 | 0.0011 | 6527 | 13641 | 3.55 | WBS |
| Metabolism; Carbohydrate metabolism | TCA cycle IV (2-oxoglutarate decarboxylase) | 16.22 | 0.0001 | 0.0008 | 5624 | 12364 | 3.53 | WBS |
| Metabolism; Carbohydrate metabolism | superpathway of Clostridium acetobutylicum acidogenic fermentation | 21.36 | 0.0000 | 0.0002 | 6939 | 13024 | 3.48 | WBS |
| Metabolism; Carbohydrate metabolism | superpathway of glycolysis, pyruvate dehydrogenase, TCA, and glyoxylate bypass | 16.75 | 0.0000 | 0.0007 | 4566 | 10351 | 3.46 | WBS |
| Metabolism; Carbohydrate metabolism | pyruvate fermentation to butanoate | 21.28 | 0.0000 | 0.0002 | 5659 | 10719 | 3.4 | WBS |
| Metabolism; Carbohydrate metabolism | succinate fermentation to butanoate | 23.24 | 0.0000 | 0.0001 | 3334 | 8234 | 3.39 | WBS |
| Metabolism; Carbohydrate metabolism | L-lysine fermentation to acetate and butanoate | 13.13 | 0.0003 | 0.0026 | 3701 | 8040 | 3.34 | WBS |
| Metabolism; Carbohydrate metabolism | superpathway of glyoxylate bypass and TCA | 16.89 | 0.0000 | 0.0007 | 3050 | 7360 | 3.33 | WBS |
| Metabolism; Carbohydrate metabolism | glyoxylate cycle | 12.9 | 0.0003 | 0.0027 | 2933 | 6414 | 3.24 | WBS |
| Metabolism; Carbohydrate metabolism | D-glucarate degradation I | 14.21 | 0.0002 | 0.0016 | 2486 | 5650 | 3.2 | WBS |
| Metabolism; Carbohydrate metabolism | D-galactarate degradation I | 15.02 | 0.0001 | 0.0011 | 1869 | 4962 | 3.19 | WBS |
| Metabolism; Carbohydrate metabolism | superpathway of D-glucarate and D-galactarate degradation | 15.02 | 0.0001 | 0.0011 | 1869 | 4962 | 3.19 | WBS |
| Metabolism; Carbohydrate metabolism | ketogluconate metabolism | 18.06 | 0.0000 | 0.0004 | 299 | 1295 | 2.7 | WBS |
| Metabolism; Carbohydrate metabolism | glucose degradation (oxidative) | 15.7 | 0.0001 | 0.001 | 53 | 479 | 2.33 | WBS |
| Metabolism; Xenobiotics biodegradation and metabolism | superpathway of glycol metabolism and degradation | 16.78 | 0.0000 | 0.0007 | 1735 | 4781 | 3.18 | WBS |
| Metabolism; Xenobiotics biodegradation and metabolism | 4-aminobutanoate degradation V | 17.62 | 0.0000 | 0.0005 | 4380 | 6811 | 3.09 | WBS |
| Metabolism; Xenobiotics biodegradation and metabolism | protocatechuate degradation II (ortho-cleavage pathway) | 18.41 | 0.0000 | 0.0004 | 178 | 942 | 2.58 | WBS |
| Metabolism; Xenobiotics biodegradation and metabolism | aromatic compounds degradation via &beta;-ketoadipate | 20.24 | 0.0000 | 0.0002 | 90 | 555 | 2.37 | WBS |
| Metabolism; Xenobiotics biodegradation and metabolism | catechol degradation III (ortho-cleavage pathway) | 20.24 | 0.0000 | 0.0002 | 90 | 555 | 2.37 | WBS |
| Metabolism; Xenobiotics biodegradation and metabolism | catechol degradation to &beta;-ketoadipate | 20.57 | 0.0000 | 0.0002 | 84 | 550 | 2.37 | WBS |
| Metabolism; Metabolism of cofactors and vitamins | superpathway of heme biosynthesis from uroporphyrinogen-III | 12.6 | 0.0004 | 0.003 | 2698 | 5299 | 3.11 | WBS |
| Metabolism; Metabolism of cofactors and vitamins | superpathway of heme biosynthesis from glycine | 15.17 | 0.0001 | 0.0011 | 1373 | 2707 | 2.82 | WBS |
| Metabolism; Metabolism of cofactors and vitamins | nicotinate degradation I | 13.31 | 0.0003 | 0.0024 | 59 | 405 | 2.24 | WBS |
| Metabolism; Glycan biosynthesis and metabolism | superpathway of (Kdo)2-lipid A biosynthesis | 16.25 | 0.0001 | 0.0008 | 3204 | 6730 | 3.25 | WBS |
| Metabolism; Glycan biosynthesis and metabolism | enterobacterial common antigen biosynthesis | 16.36 | 0.0001 | 0.0008 | 1236 | 3478 | 3.05 | WBS |
| Metabolism; Lipid metabolism | fatty acid &beta;-oxidation I | 23.74 | 0.0000 | 0.0001 | 4415 | 10535 | 3.49 | WBS |
| Metabolism; Lipid metabolism | glutaryl-CoA degradation | 15.27 | 0.0001 | 0.0011 | 3224 | 5541 | 3.06 | WBS |
| Metabolism; Metabolism of terpenoids and polyketides | enterobactin biosynthesis | 15.23 | 0.0001 | 0.0011 | 2590 | 5287 | 3.13 | WBS |
| Metabolism; Biosynthesis of other secondary metabolites | aerobactin biosynthesis | 14.69 | 0.0001 | 0.0013 | 6 | 96 | 1.66 | WBS |
| Cellular Processes; Cellular community - prokaryotes | ppGpp biosynthesis | 19.69 | 0.0000 | 0.0002 | 2920 | 6434 | 3.25 | WBS |
| Environmental Information Processing; Signal transduction | polymyxin resistance | 18.09 | 0.0000 | 0.0004 | 983 | 3222 | 3.05 | WBS |


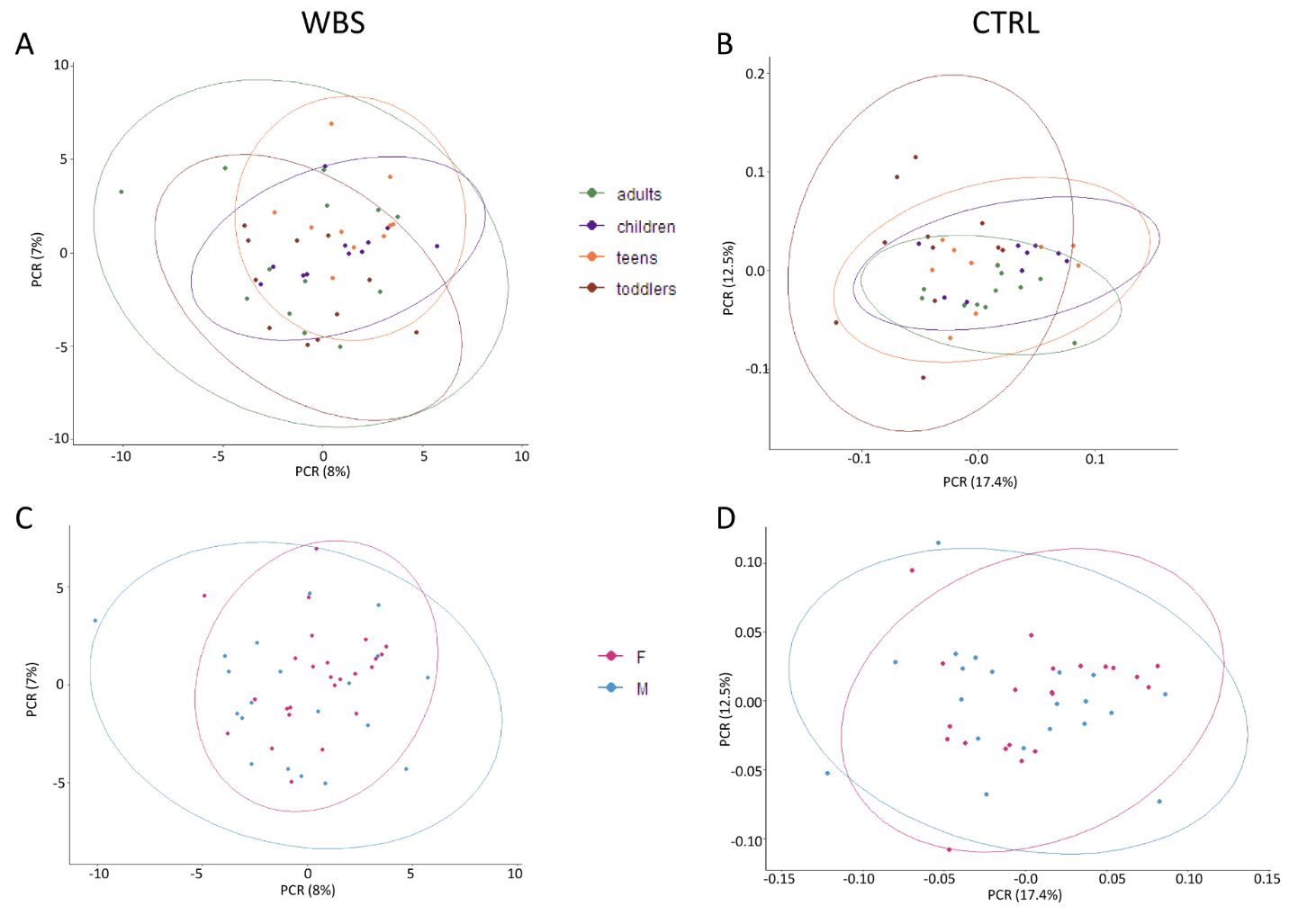


**Supplementary Figure S1. Unsupervised Principal Component Analysis (PCA).** 2D PCA-plots showing the clustering of WBS (A-C) and CTRL (B-D) samples labelled for class age and gender, respectively.


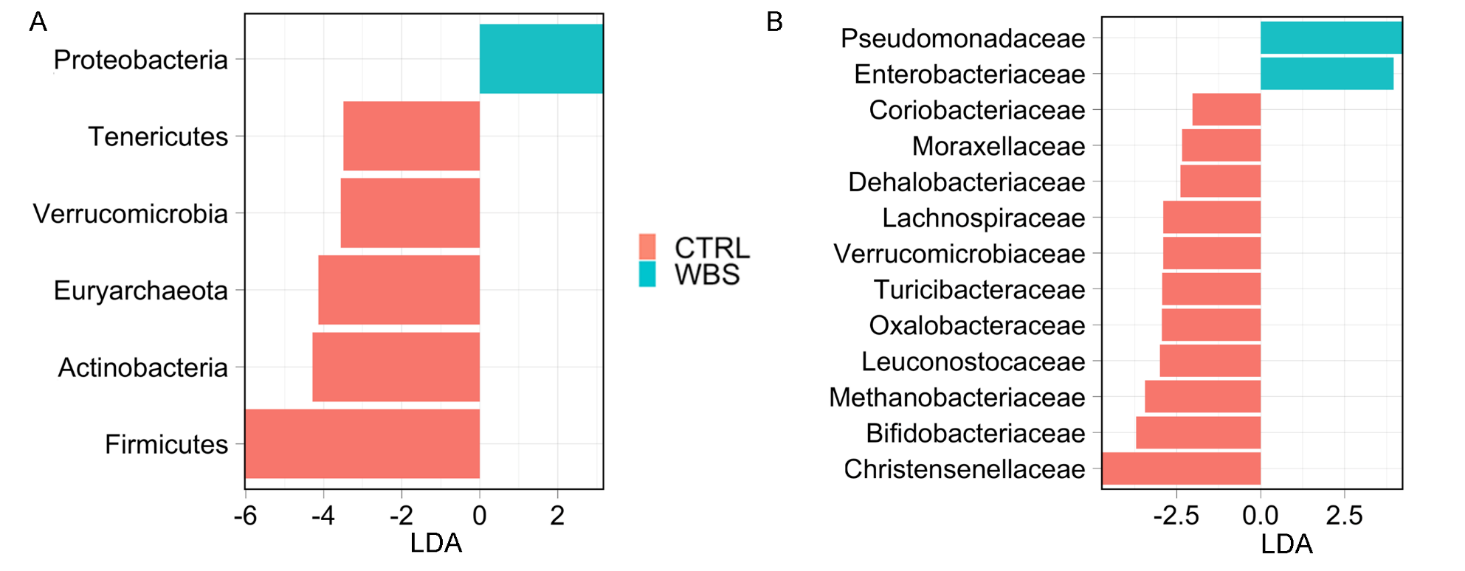


**Supplementary Figure S2. Differential abundance analysis performed by ANCOM-BC (analysis of compositions of microbiomes with bias correction) test** at **A**) phylum level and **B**) family level. The plots show fold change of differential abundant bacteria between WBS and CTRL. The comparisons of bacterial abundances between WBS and CTRL revealed the increase in Proteobacteria, and the decrease in Tenericutes, Verrucomicrobia, Euryarcheota, Actinobacteria, and Firmicutes in WBS (**Panel A; Table S1**). At family level, Pseudomonadaceae, Enterobacteriaceae were increased, and Coriobacteriaceae, Moraxellaceae, Dehalobacteriaceae, Lachnospiraceae, Verrucomicrobiaceae, Turicibacteriaceae, Oxalobacteriaceae, Leuconostocaceae, Methanobacteriaceae, Bifidobacteriaceae, Christenesenellaceae were decreased in WBS (**Panel B; Table S1**).


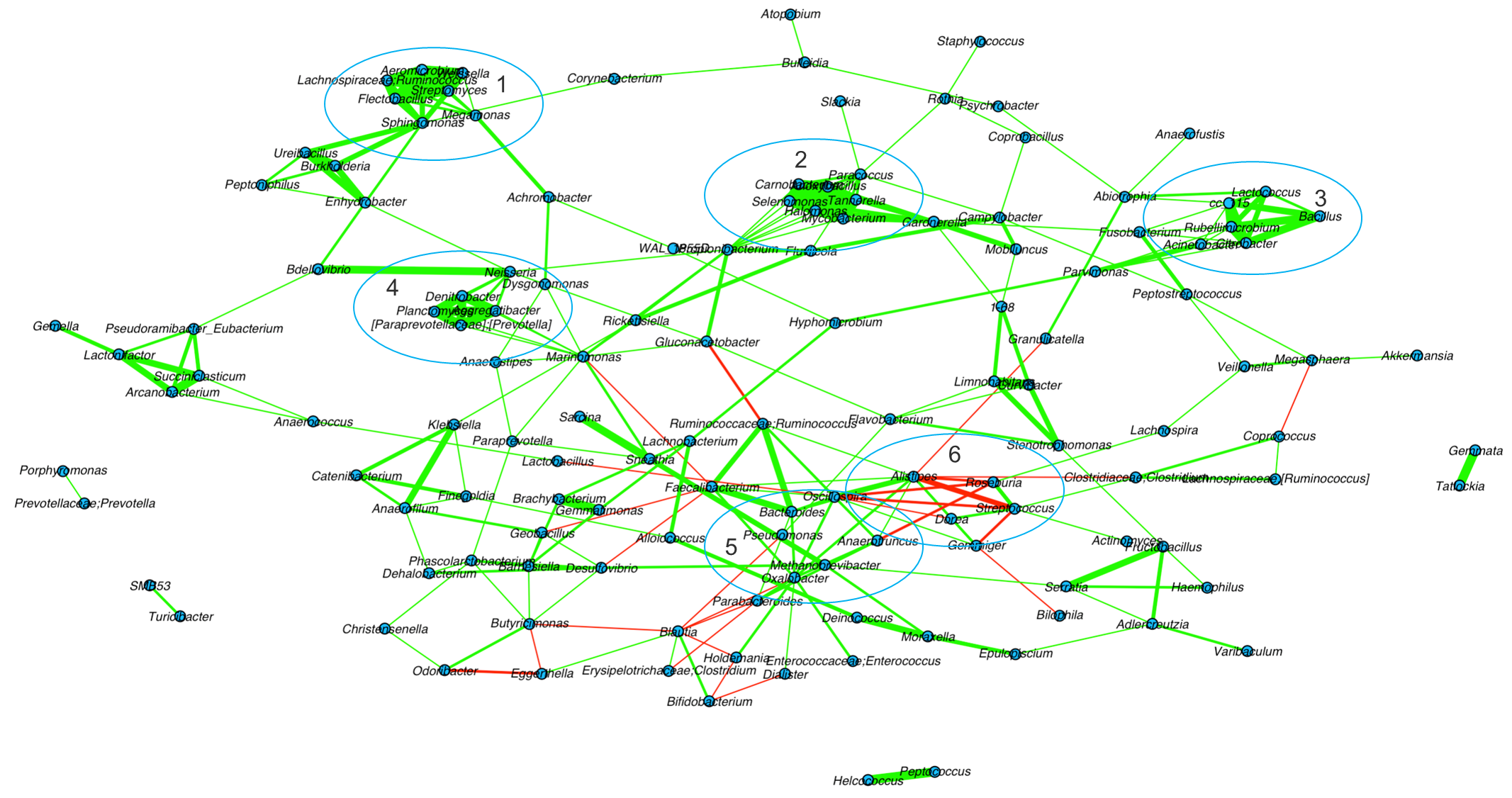


**Supplementary figure S3. Bacterial correlation network in WBS.** Correlation networks between bacteria based on Spearman’s correlation matrix. Each node (blue circles) represents the ASVs at genus level. Green and red edges, indicate positive and negative correlation values, respectively. Only correlations statistically significant (p-value <0.05) are represented.

In WBS we highlighted 6 major networks. In the first, *Sphingomonas* was positively linked to *Megamonas, Flectobacillus, Streptomyces, Ruminococcus* (Lachnospiraceae)*, Aeromicrobum, Weissella, Ureibacillus, Burkholderia* and *Enhydrobacter*. In the second, *Paracoccus* was positively correlated with *Slackia, Rothia, Campylobacter, Tannarella, Carnobacterium, Selenomonas, Halomonas, Mycobacterium* and *Fluviivola*, the last 5 taxa were also connected with *Propionibacterium*. The third network was composed by the interconnection amongst *Lactococcus, Bacillus, Citrobacter, Acinetobacter, Rubellimicrobium, cc_115* and *Parvimonas*. The fourth network was composed by the interconnection of *Neisseria*, *Denitrobacter*, *Aggregatibacter*, *Planctomyces*, *Prevotella* (Paraprevotellaceae). The fifth network linked *Oxalobacter*, *Methanobrevibacter*, *Anaerotruncus*, *Pseudomonas*, *Alistipes*, *Oscillospira*, *Bacteroides*, *Faecalibacterium*, *Parabacteroides*, *Holdemania*, *Enterococcus* (Enterococcaceae) and *Blautia* (negative correlation). Finally, in the sixth, *Alistipes* was positively connected with *Faecalibacterium*, *Oscillispira*, *Anaerotruncus*, *Gemmiger* and negatively with *Granulicatella*, *Clostridium* (Clostridiaceae), *Roseburia* and *Streptococcus.*


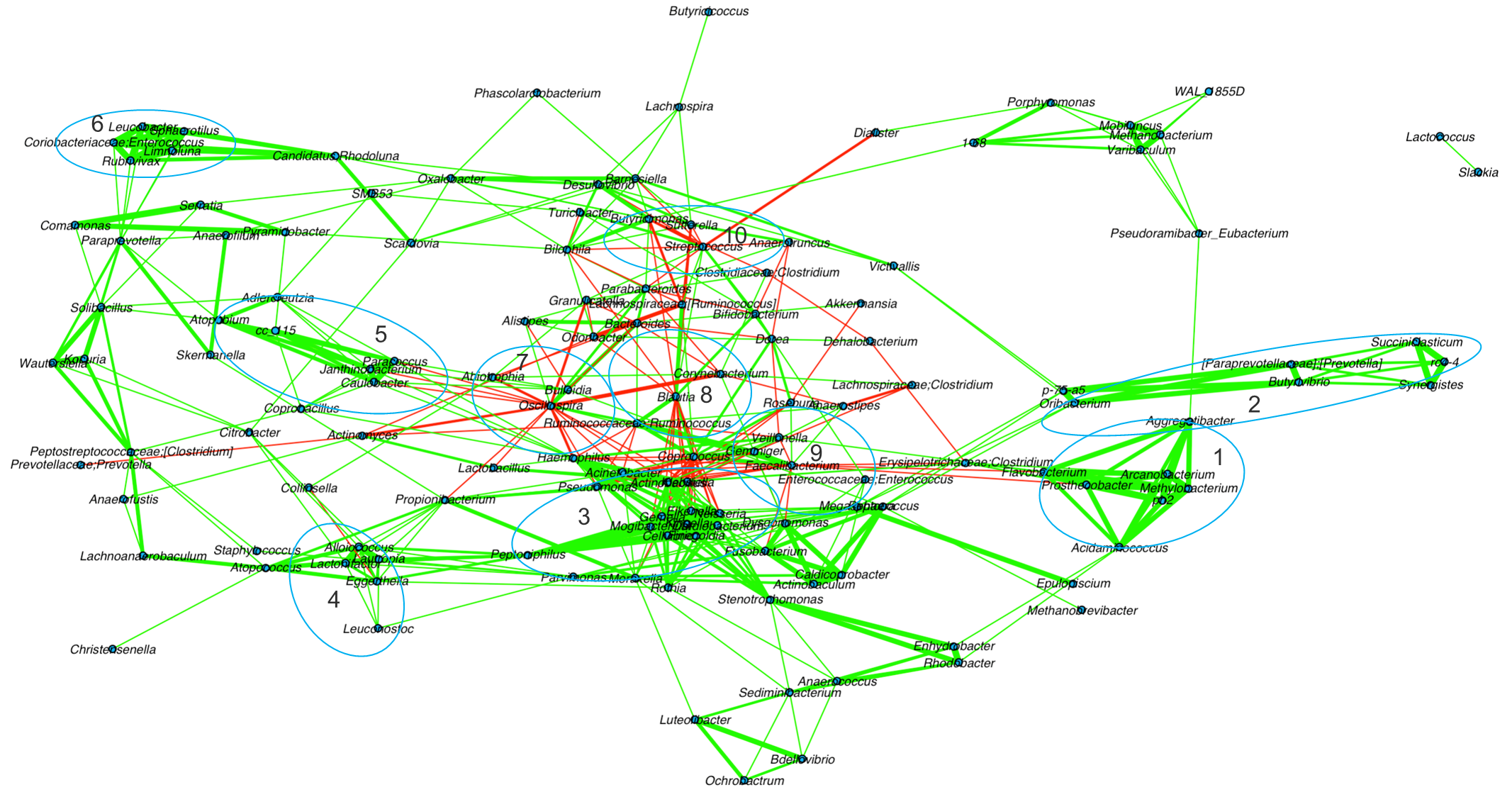


**Supplementary Figure S4. Bacterial correlation network of CTRL.** Correlation networks between bacteria based on Spearman’s correlation matrix. Each node (blue circles) represents the ASVs at genus level. Green and red edges, indicate positive and negative correlation values, respectively. Only correlations statistically significant (p-value <0.05) are represented.

In CTRLs we have identified 10 major nodes. In the first, *Aggregatibacter* and *Acidaminococcus* were linked to *Flavobacterium*, *Prostheobacter*, *Arcanobacterium*, *Methylobacterium*, ph2 and *Acidaminococcus*. In the second one, *Prevotella* (Prevotellaceae), *Succiniclasticum,* *Synergistetes* and *Butyrivibrio* were linked together. In the third node, *Neisseria*, *Eikenella*, *Gemella*, *Mogibacterium* and *Finegoldia* were positively connected with *Stenotrophomonas*, *Rothia*, *Peptoniphilus* and *Megasphera*, and negatively with *Blautia*. The fourth node was composed by *Leuconostoc*, *Eggerthella*, *Lautropia*, *Alloicoccus* *Atopococcus*, *Lactonifactor*, *Propionibacterium* and *Parvimonas*. The fifth one was composed by *Paracoccus*, *Janthinobacterium* *Caulobacter*, *Coprobacillus* and *Atopobium*. The sixth was composed by *Candidatus Rhodoluna* and *Paraprevotella* linked with *Leucobacter*, *Spherotilus,* *Enterococcus* (Coriobacteriaceae), *Limnluna* and *Rubrivivax*. The seventh one was composed by *Oscillospira* negatively linked to *Corynebacterium*, *Acinetobacter*, *Pseudomonas*, *Actinobacillus*, *Klebsiella,* *Haemophilus*, *Lactobacillus*, *Propionibacterium,* *Actinomyces,* *Caulobacter*, *Janthinobacterium*, *Paracoccus,* *Abiotrophia,* *Granulicatella* and positively with *Alistipes*, *Bacteroides* and *Parabacteroides*. In the eighth node, *Blautia* was negatively correlated with *Odoribacter*, *Bilophila*, *Butyricimonas*, *Mogibacterium*, *Gemella*, *Cellvibrio*, *Finegoldia* *Coprococcus* and *Neisseria* and positively with *Ruminococcus* (Ruminococcaceae), *Bifidobacterium* and *Ruminococcus* (Lachnospiraceae). In the ninth node, *Faecalibacterium* was negatively correlated with *Clostridium* (Erysipelotrichaceae), *Flavobacterium*, *Prosthecobacter*, *Veillonella*, *Acinetobacter*, *Pseudomonas*, *Actinobacillus*, *Klebsiella,* *Haemophilus, Dysgonomonas* and *Fusobacterium,* while positively with *Roseburia, Anaerostipes* and *Gemmiger.* Finally, in the tenth node, *Streptococcus* was negatively linked to *Dialister, Anaerotruncus, Clostridium* (Clostridiaceae), *Parabacteroides, Bilophila, Butyricimonas, Desulfovibrio, Barnesiella, Sutterella*, while positively with *Bifidobacterium, Balutia, Ruminococcus* (Lacnospiraceae), *Granulicatella* and *Turicibacter.*


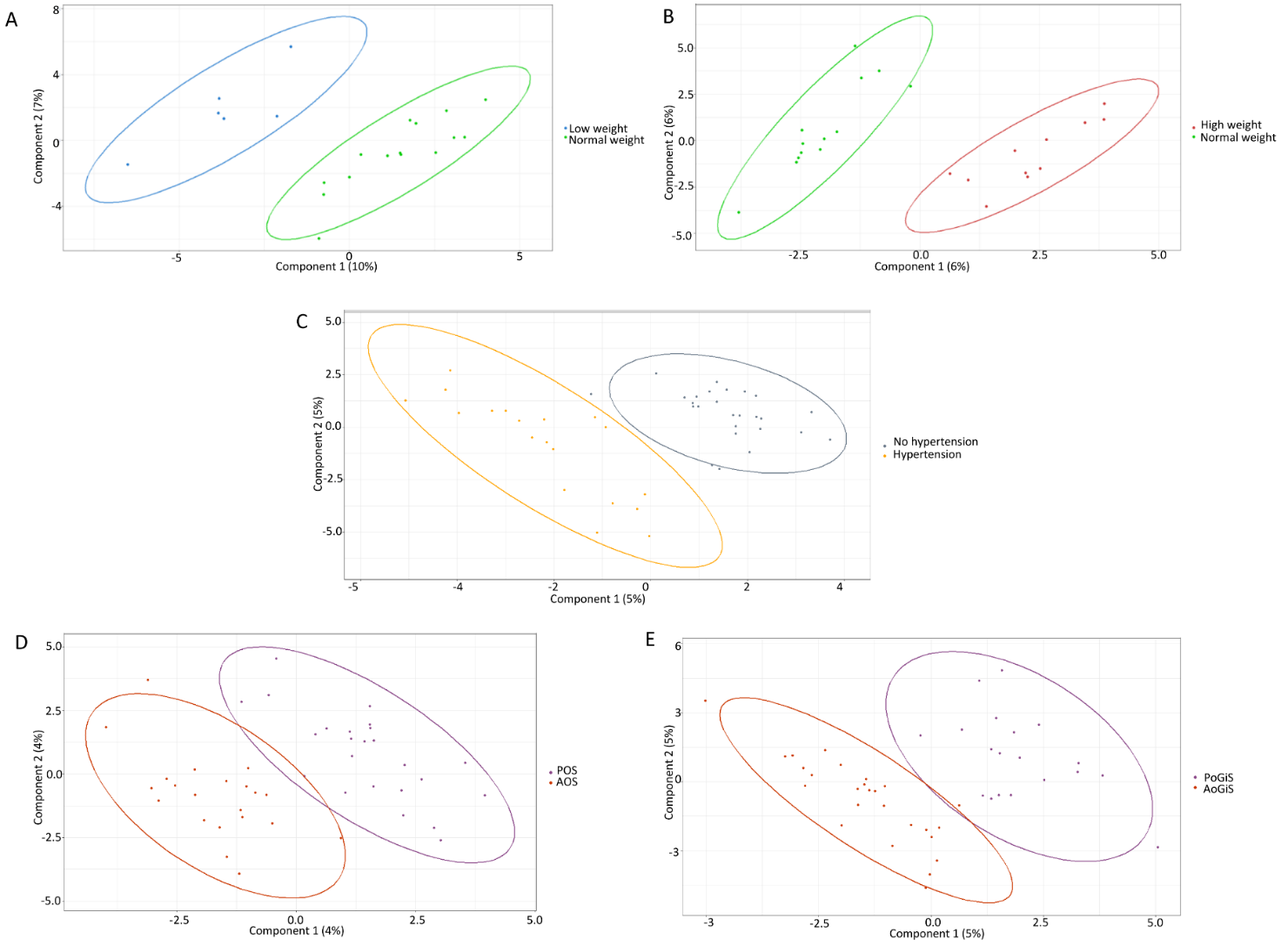


**Supplementary Figure S5. Partial Least Squares-Discriminant Analysis (PLS-DA) plots of the WBS dataset classified for weight‐related and clinical features.** Panel A, low weight and normal weight, Q^2^=-0.217, Root Mean Square Error (RMSE) = 0.125; Panel B, high weight and normal weight, Q^2^=-0.062, RMSE=0.111; Panel C, no hypertension and hypertension, Q^2^=-0.030, RMSE=-0.185; Panel D, absence (AoGiS) and presence of gastrointestinal symptoms (PoGiS), Q^2^=-0.031, RMSE=0.193; absence (AOS) and presence of at least 1 gastrointestinal symptom (POS), Q^2^=-0.154, RMSE=0.207
